# Recent trends in multiple metrics and multimodal analysis for neural activity and pupillometry

**DOI:** 10.3389/fneur.2024.1489822

**Published:** 2024-12-02

**Authors:** Sou Nobukawa, Aya Shirama, Tetsuya Takahashi, Shigenobu Toda

**Affiliations:** ^1^Department of Computer Science, Chiba Institute of Technology, Narashino, Chiba, Japan; ^2^Graduate School of Information and Computer Science, Chiba Institute of Technology, Narashino, Chiba, Japan; ^3^Research Center for Mathematical Engineering, Chiba Institute of Technology, Narashino, Chiba, Japan; ^4^Department of Preventive Intervention for Psychiatric Disorders, National Institute of Mental Health, National Center of Neurology and Psychiatry, Tokyo, Japan; ^5^Research Center for Child Mental Development, Kanazawa University, Kanazawa, Japan; ^6^Department of Neuropsychiatry, University of Fukui, Fukui, Japan; ^7^Uozu Shinkei Sanatorium, Uozu, Toyama, Japan; ^8^Department of Psychiatry, Shizuoka Psychiatric Medical Center, Shizuoka, Japan; ^9^Department of Psychiatry and Behavioral Science, Kanazawa University, Kanazawa, Japan; ^10^Department of Psychiatry, Showa University, Tokyo, Japan

**Keywords:** cognitive function, complexity, emergence, graph analysis, functional connectivity, neuroimaging, multimodal data, pupillometry

## Abstract

Recent studies focusing on neural activity captured by neuroimaging modalities have provided various metrics for elucidating the functional networks and dynamics of the entire brain. Functional magnetic resonance imaging (fMRI) can depict spatiotemporal functional neural networks and dynamic characteristics due to its excellent spatial resolution. However, its temporal resolution is limited. Neuroimaging modalities such as electroencephalography (EEG) and magnetoencephalography (MEG), which have higher temporal resolutions, are utilized for multi-temporal scale and multi-frequency-band analyzes. With this advantage, numerous EEG/MEG-bases studies have revealed the frequency-band specific functional networks involving dynamic functional connectivity and multiple temporal-scale time-series patterns of neural activity. In addition to analyzing neural data, the examination of behavioral data can unveil additional aspects of brain activity through unimodal and multimodal data analyzes performed using appropriate integration techniques. Among the behavioral data assessments, pupillometry can provide comprehensive spatial-temporal-specific features of neural activity. In this perspective, we summarize the recent progress in the development of metrics for analyzing neural data obtained from neuroimaging modalities such as fMRI, EEG, and MEG, as well as behavioral data, with a special focus on pupillometry data. First, we review the typical metrics of neural activity, emphasizing functional connectivity, complexity, dynamic functional connectivity, and dynamic state transitions of whole-brain activity. Second, we examine the metrics related to the time-series data of pupillary diameters and discuss the possibility of multimodal metrics that combine neural and pupillometry data. Finally, we discuss future perspectives on these multiple and multimodal metrics.

## 1 Introduction

Mounting evidence from recent studies focusing on neural activities captured by neuroimaging modalities has provided diverse metrics for elucidating functional networks and dynamics in the entire brain [reviewed in Sporns and Seguin et al. (1, 2)]. The analysis of functional magnetic resonance imaging (fMRI) data to ascertain functional connectivity facilitates the elucidation of the functional whole brain network, called the “functional connectome” (3–5), owing to its fine spatial resolution for the brain, including the deep-located nuclei. Moreover, functional networks show dynamic (called dynamic functional connectivity) rather than static characteristics in response to cognitive and perceptual stimuli, even during the resting state (6–8) [reviewed in Preti et al. (9)]. This dynamic organization of functional connectivity is strongly linked to the complexity of local regional neural activity (10). Brain function is a representative “emergence” phenomenon produced by the integration of hierarchical and mutual interactions of neural activities of the brain (11–13). Notably, the broad-range mutual interactions among neural activities enable cognitive functions as the most complex emergent phenomena, venturing beyond mere signal propagation between specific regions [as reviewed in Thiebaut de Schotten and Forkel (14)]. To elucidate this mechanism, the use of comprehensive metrics of whole-brain spatiotemporal neural activities has gained widespread recognition (1).

**Figure 1:**
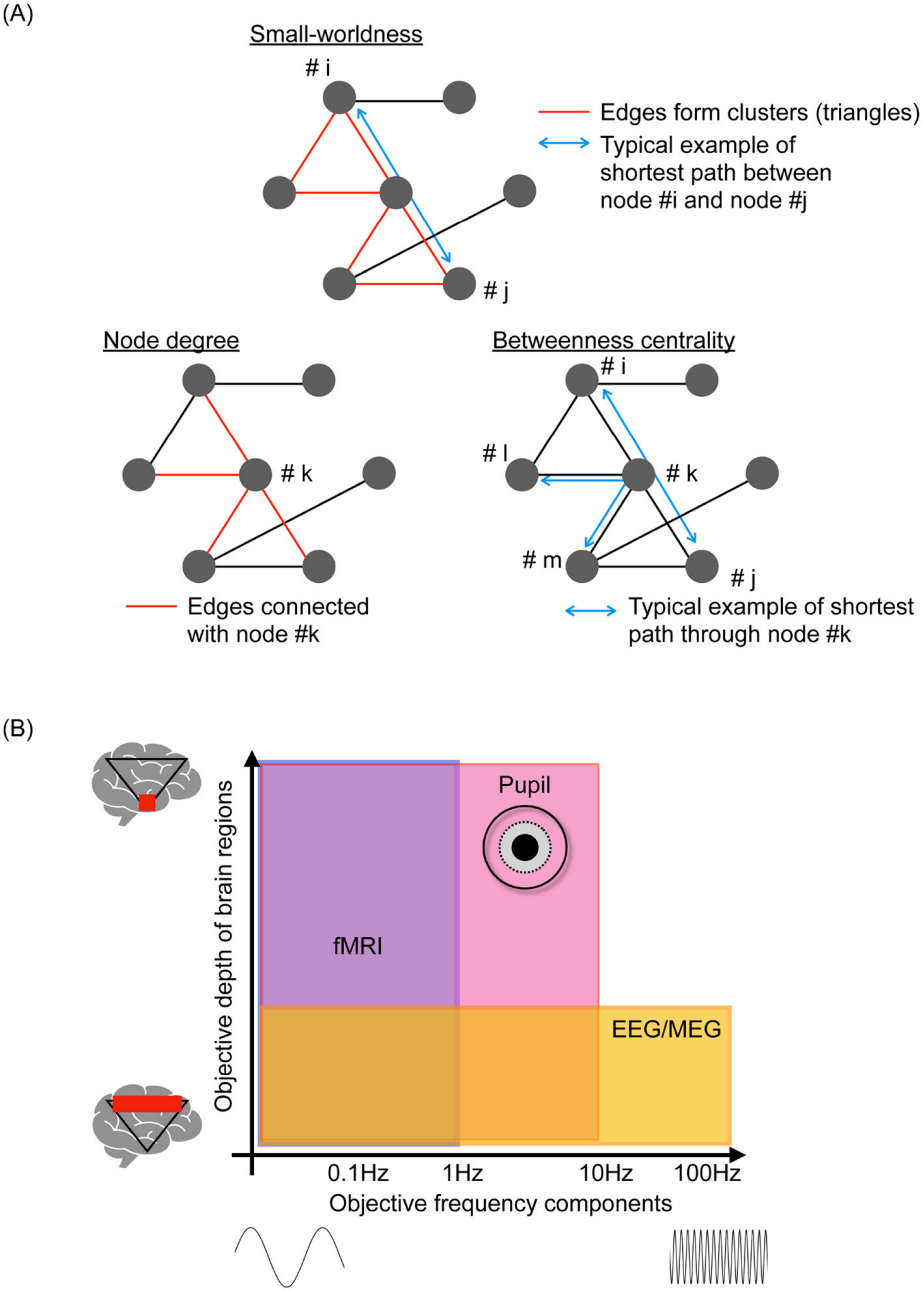
**(A)** Typical examples of metrics as a topological feature of functional networks. Small-worldness based on the ratio of the clustering coefficient and path length, which represents the segregation and efficiency of signal propagation within the functional networks. Hub characteristics such as node degree (edge numbers in the node) and betweenness centrality (number of shortest paths passing through the node), which are effective for evaluating integration and propagation within functional networks. **(B)** Spatial-temporal objective domain of representative neuroimaging, including functional magnetic resonance imaging (fMRI), electroencephalography (EEG), and magnetoencephalography (MEG), pupillometry.

fMRI can depict spatiotemporal functional neural networks and dynamic characteristics due to its excellent spatial resolution. However, its temporal resolution is limited ≲1 Hz. This prevents the elucidation of neural activity characteristics over a wide frequency range including delta, theta, alpha, beta, gamma, and high gamma ≲100 [Hz] and moment-to-moment dynamics (15). Neuroimaging modalities such as electroencephalography (EEG) and magnetoencephalography (MEG) with higher time resolutions have been utilized for multi-temporal scale and multi-frequency-band analyzes [reviewed in Beppi et al., Iivanainen et al., and Niso et al. (16–18)]. The vast collection of EEG/MEG data in these studies revealed the frequency-band-specific functional network involving dynamic functional connectivity and multiple temporal-scale time-series patterns of neural activities. This was captured as “complexity,” which refers to the degree of irregularity observed in time-series data of neural activity, evaluated using metrics from non-linear time-series analysis, including chaos theory, fractal analysis, and various types of entropy measures; additionally, moment-to-moment dynamic state transitions based on whole-brain neural activity were analyzed to capture temporal dynamics (19–22). However, these neuroimaging modalities have limitations related to volume conduction, which refers to the spurious synchronization of regional neural activities (23, 24). By focusing on the phase components of neural activities, this influence can be curtailed and typified as the phase lag index (PLI) and weighted PLI (25–29). Subsequently, the combinations of high-density EEG and MEG data and complementary cortical source localization techniques have significantly augmented the spatial resolution, yielding sustained high temporal resolutions (30–33). Moreover, considering the assembly of functional connectivity as a topological feature enables assessment of the functional networks among widely distributed brain regions [reviewed in Farahani et al., Ismail and Karwowski, Pegg et al. (34–36); see the typical example of Figure 1A).

In addition to the analysis of neural data, the examination of behavioral data, such as heart rate, body movements, eye movements, and pupil behaviors, can reveal additional characteristics of brain activity through unimodal [reviewed in Lujan et al., Mahanama et al., and Gullett et al. (37–39)] and multimodal data analyses with appropriate integration techniques [reviewed in Iwama et al. (40)]. This encompasses daily fluctuations in neural activity, known as the circadian rhythm [reviewed in Foster (41)], the balance between neural systems in whole-brain activity, such as the sympathetic and parasympathetic neural systems [reviewed in Hyun and Sohn (42)], and the activity patterns of deep-located neural nuclei [reviewed in Aston-Jones and Cohen and Viglione et al. (43, 44)]. Amongst these behavioral data, pupillometry can provide comprehensive spatial-temporal-specific features of neural activity (45–47). Figure 1B illustrates the relationship between the depth of signal-source location in the brain and the major frequency components of the time-series data obtained by each neuroimaging modality. Pupillometry detects relatively high-frequency components of neural activity, ≲ several Hz, in deep-located brain regions; fMRI and EEG/MEG cannot capture such high-frequency components and the neural activities of deep brain regions (45, 48). Leveraging this specificity can pave the way for understanding the mechanisms underlying brain functions and introduce a novel approach for identifying dysfunctions responsible for psychiatric disorders (49). Effectively ascertaining the dynamic patterns of neural activity using pupillometry is beset by challenges when extremely high-dimensional brain activities are projected onto two-dimensional time series of pupil diameters in the left and right eyes through signal propagation with nonlinear transformation and diverse interactions of inherent neural activities (50, 51). Therefore, developing metrics to estimate the internal neural activity from the time-series data of pupil diameters is important, as evidenced by the substantial focus of recent research (49). Moreover, although many recent multimodal studies focused primarily on pupil size (52, 53), there have been increasing attempts to capture innate neural activity based on pupil dynamics beyond simple size measurements (51, 54–56).

In this perspective, we summarize the recent progress in developing metrics for analyzing neural data obtained from neuroimaging modalities such as fMRI, EEG, and MEG as well as behavioral data, with a focus on pupillometry data. First, we review the typical metrics of neural activity, emphasizing functional connectivity, complexity, dynamic functional connectivity, and dynamic state transitions of whole-brain activity. Second, we examine the metrics related to the time-series data of pupil diameters. Third, we discuss the possibility of multimodal metrics that combine neural and pupillometry data. Finally, we discuss future perspectives on these multiple and multimodal metrics.

## 2 Analysis of neural data

In the classical interpretation of cognitive functions, individual brain regions are viewed as modular systems, each playing a specific cognitive role. Information processed in each region is subsequently propagated through hierarchical neural pathways. However, recent neuroimaging studies have revealed that cognitive functions are the result of interactions among widely distributed brain regions, rather than mere signal propagation between specific regions [reviewed in Thiebaut de Schotten and Forkel (14)].

To elucidate local and regional neural activities, the levels of blood oxygen level-dependent (BOLD) signals in fMRI and the power components of EEG/MEG signals continue to be widely utilized, serving as the initial foundational methods. Moreover, functional connectivity is considered the most representative approach to evaluate signal propagation and the integration of brain activities (3–5). Initially, this evaluation employs correlation coefficients and coherence measures based on cross-spectrum analysis, which are used in fMRI. However, for EEG/MEG signals, the influence of volume conduction, which causes spurious synchronization due to the observation of the same signal source at different sensors on the scalp, degrades the spatial resolution of functional networks (23, 24). This influence can be mitigated by metrics that focus on phase components, such as the PLI (57) and the weighted PLI, an enhanced iteration of the PLI (27). By leveraging the high temporal resolution of EEG/MEG combined with these improvements, these metrics have been utilized to delineate frequency-band-specific functional networks related to aging (58), various cognitive functions (59), and psychiatric disorders (60, 61).

In addition to evaluating functional connectivity based on mere pairwise neural activities, assessing the interactions of neural activity at the whole-brain level entails considering the assembly of functional connectivity as a topological feature of functional networks [see the overview of typical example of network measures in Figure 1A; reviewed in Farahani et al., Ismail and Karwowski, and Pegg et al. (34–36)]. The clustering coefficient, path length, and their ratio, which are measures of small-worldness, represent the segregation and efficiency of signal propagation within the functional networks (62–64). Hub characteristics such as node degree and betweenness centrality (65) are effective for evaluating the integration and propagation within these networks. These metrics can capture aspects of functional network topology in cognitive processes (32), their abilities (66), and psychiatric disorders (67, 68). These topological features reflect the complexity of time-series data pertaining to local and regional activities. Specifically, the hub region of the brain is influenced by other brain regions during the integration process; this interaction induces region-specific complexity (58, 69, 70). This characteristic is significantly advantageous from an application viewpoint because the local and regional complexity can be evaluated using low-density EEG equipment, which offers high versatility. High-density EEG is required for evaluating the topological features of functional networks (71).

In the interactions among brain regions, the evaluation of functional networks based on averages obtained over long time-windows, known as static functional connectivity, is not sufficient. The interaction of neural activity during cognitive processes, even during the resting state, exhibits significant dynamic properties [reviewed in Hutchison et al. (72)]. Dynamic functional connectivity, which involves the temporal variation of functional connectivity through sliding windows, was proposed to evaluate dynamic interactions [reviewed in Hutchison et al. (72)]. This approach to dynamic functional connectivity has succeeded in capturing the network dynamics that support cognitive functions (73, 74) and alterations caused by psychiatric disorders (75, 76). Historically, there was ambiguity in setting the window, but currently, by associating it with the distribution of quasi-stable states of neural activity, a strict window setting can be achieved, allowing this method to successfully detect network patterns that reflect cognitive processes (77). Another recent advancement in studies on dynamic functional connectivity is the focus on dynamic phase synchronization instead of mere synchronization. This emphasizes the patterns produced by the phase difference in instantaneous phase components of neural activities between brain regions, and has successfully detected age-related alterations in functional networks, representing a significant improvement in detection abilities (78). Dynamic phase synchronization has evolved into a novel method for detecting dynamic whole-brain activity states based on the whole-brain distribution of instantaneous frequency, termed instantaneous frequency micro-states (79).

Considering future research trends for metrics of neural data, novel metrics are needed instead of focusing solely on the synchronization of neural activity. This is because the neural activity of the brain is dynamic in nature, including transient behaviors (79, 80). Therefore, the assumption of time invariance during certain periods in the evaluation of synchronization proves difficult in the presence of moment-to-moment dynamic behaviors. A recent study revealed that the propagation of a momentary drop in complexity within local and regional neural activity modulates interactions in hierarchical neural networks (10). This implies that moment-to-moment dynamic characteristics, which cannot be captured by synchronization alone, are essential for precise interactions related to brain activity. Metrics that address this challenge are likely to be developed in the future.

## 3 Analysis of pupil data

### 3.1 Metrics for pupillometry

**Figure 2:**
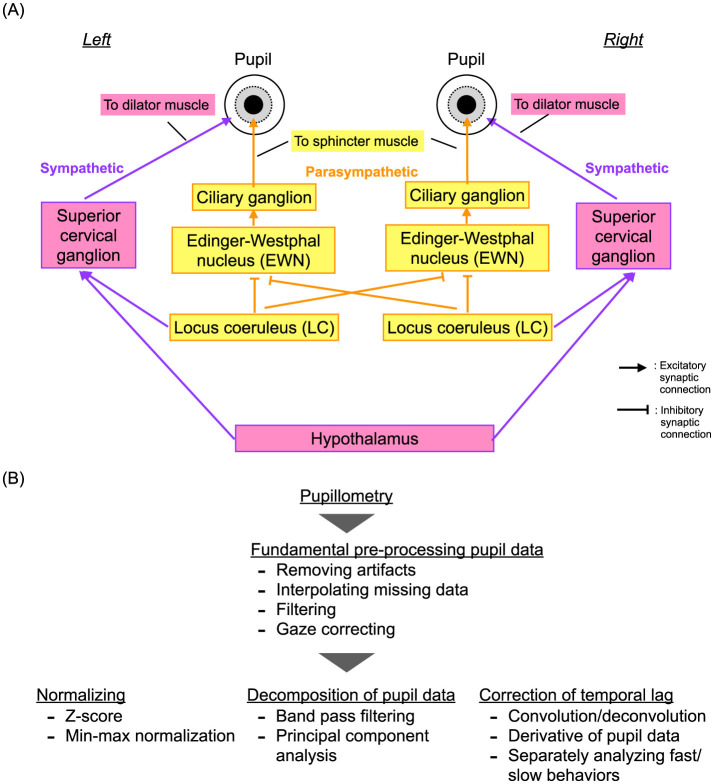
**(A)** Neural pathways for regulating pupil diameter: the dilator muscle controlled by the sympathetic neural system and the sphincter muscle governed by the parasympathetic neural system. **(B)** Typical preprocessing pipeline for pupil data, including artifact removal, normalization, and decomposition.

In 1982, Usui and Stark demonstrated an inverted *U*-shape profile of the temporal complexity of pupil diameter characteristics relative to the degree of dilation (50). They attributed this phenomenon to autonomous fluctuations in internal neural activity and the non-linearity of neural pathways that control pupil diameter in response to the levels of activity. This understanding was based on the erstwhile knowledge that pupil diameters are regulated by the dilator muscle controlled by the sympathetic neural system and the sphincter muscle controlled by the parasympathetic neural system. Recently, it has been discovered that the autonomous neural fluctuations originate from the locus coeruleus (LC), which serves mainly as a common source for the sympathetic and parasympathetic pathways, at least partially (81) (see the overview of these neural pathways in Figure 2A). The LC plays a crucial role in coordinating the arousal and attention functions [reviewed in Aston-Jones and Cohen (43)]. Specifically, the inhibitory projections from the LC to the Edinger-Westphal nucleus (EWN) include contralateral projections on the left and right sides, and not only ipsilateral projections. This is unlike the sympathetic neural pathway, which comprises only ipsilateral projections (82). Furthermore, the LC, other nuclei, and various brain regions are sources of fluctuations in neural activity that significantly influence pupil diameter behaviors, depending on the cognitive process. This influence has been demonstrated by multimodal experiments that combined pupillometry with fMRI (46, 83). These characteristics of pupil diameters involve neural activity deep within the brain that cannot be captured by EEG/MEG (43) and faster components (45, 48) that cannot be detected by fMRI. Therefore, the addition of pupillometry is crucial for capturing deep brain interactions. However, pupil data present unique challenges due to missing data and artifacts from gaze movements and blinks, as well as nonlinear transformations and multiple projections from various brain regions and nuclei. Despite a substantial body of research proposing fundamental preprocessing, normalization, decomposition techniques, and temporal lag correction for pupil response [as illustrated in Figure 2B and reviewed in Fink et al. and Shirama et al. (84, 85)], evaluating such complex pupil data remains a challenging process.

In addition to assessing pupil size, which is the focus of the majority of studies (43, 46, 83, 86), the application of non-linear dynamic analysis has also progressed (51, 54–56). Recent studies have identified deterministic properties of pupil behavior that reflect internal neural activity (51, 55). Nobukawa et al. revealed the deterministic properties of spontaneous pupil behavior by combining sample entropy and surrogate data analyzes. Sviridova et al. also demonstrated these deterministic properties using various types of nonlinear analyzes, such as metrics from recurrence plots, the largest Lyapunov exponent, and deterministic nonlinear prediction (55). These properties, characterized by the complexity and symmetry of the left and right pupil diameters, are indicative of sleepiness (54) and deficits in attentional function (49, 56). Specifically, the complexity measured by Shannon entropy and determinism in cross-recurrence analysis correlates with sleepiness (54). Asymmetry, measured by transfer entropy between the left and right time series of pupil size, and differences in complexity between the right and left eyes measured by sample entropy, reflect the pathology of attention deficit hyperactivity disorder [ADHD; (49, 56)]. These metrics, including pupil size, complexity, and symmetry, have a complementary relationship in identifying pathologies related to attention deficits (49); therefore, they may be utilized to derive components of neural activity from multiple brain regions and nuclei.

### 3.2 Computational models for pupil data

Modeling that incorporates non-linear neural pathways and internal neural activity reproduced by non-linear dynamic systems is effective as a complementary approach for estimating internal neural activity from pupil characteristics (50, 51). A pioneering study in this field by Usui and Stark applied fluctuating neural activity to the sympathetic (to the pupil dilator muscle) and parasympathetic pathways (to the pupil sphincter muscle) (50). In this model, the non-linear characteristics of these neural pathways elicit the emergence of detailed time-series patterns of internal neural activity near threshold levels. Consequently, the degree of temporal complexity of pupil size exhibits an inverted U-shaped relationship with the degree of dilation. This characteristic aligns well with the actual temporal behaviors of pupil size. Johansson and Balkenius developed a more complex model that considers multiple projections related to pupil properties and a neural system comprising the amygdala, LC, cerebellum, and other regions (87). Additionally, Nobukawa's model, which includes precise mapping of both contralateral and ipsilateral projections from the LC to the EWN, demonstrates that contralateral projections play a significant role in enhancing the inverted-U shaped profile of complexity for the pupillary characteristics relative to the pupil size (51). This model also successfully estimated the imbalance of internal LC activity between the left and right sides in cases of ADHD (56). Poynter suggested that pupil diameter asymmetry could reflect a left-right imbalance in LC activity, and that this degree of asymmetry is correlated with the severity of inattention, impulsivity, and hyperactivity (88). Nobukawa's model identified that hyperactivity on the right side of the LC causes the pupil diameter asymmetry observed in patients with ADHD, as demonstrated via comparisons with physiological pupil behaviors (56).

Thus, the combination of multiple metrics for pupil behavior and the application of models to neural systems controlling pupil behavior can enhance the spatiotemporal utility for estimating inherent neural activity. It can also provide complementary data obtained from neural activities and facilitate the effective integration of neural and behavioral data.

## 4 Multimodal analysis for neural and pupillometry sata

Recent studies utilizing simultaneous multimodal measurements of neural and behavioral activities, including pupil diameter, heart rate, and body movements, have demonstrated strong correlations between dynamic neural processes across various hierarchical levels, from single-neuron spikes to global brain networks, and these behavioral metrics (89–91) [see reviews in Aston-Jones and Cohen and van der Wel and van Steenbergen (43, 92)]. Specifically, the dynamic characteristics of pupil size offer valuable insights into neural states, reflecting factors such as arousal levels (93), cognitive functions (94, 95), and psychiatric conditions (49, 96–98) [reviewed in Aston-Jones and Cohen and van der Wel and van Steenbergen (43, 92)] (51).

As discussed in Section 2, various metrics have been developed to capture neural network activities. In parallel, research integrating multimodal measurements, such as the simultaneous monitoring of pupil dynamics with neuroimaging, has advanced significantly (52, 53, 99). As illustrated in Figure 1B, this approach seeks to utilize the spatial and temporal coverage of pupil measurements to complement the spatiotemporal range of EEG, MEG, and fMRI. Specifically, studies have highlighted correlations between pupil size and steady-state response BOLD signals obtained from fMRI, as well as associations with the selective attention, salience, error-detection, and decision-making processes (52). Furthermore, functional connectivity analyzes have revealed correlations between the functional connections of the superior frontal gyrus and pupil size, suggesting that these measurements can provide insights beyond regional activity alone (52).

Despite these advances, most evaluations based on multimodal measurements with pupillometry predominantly focus on pupil size (52, 53). To fully exploit the relatively high temporal resolution of pupil measurements, future research should aim to enhance the utility of indices based on pupil dynamics, as discussed in Section 3. This will help establish pupil measurements as a more integral tool complementary to multimodal neuroimaging.

## 5 Conclusion

This perspective highlighted recent advances in the study of neuroimaging and behavioral data, with a focus on pupillometry. The targeted spatiotemporal scales vary depending on the modalities used, as also on the segregation and interactions of different levels of neural activity. Future research is expected to place greater emphasis on interactions within deep brain regions that involve a broad range of fast temporal scales, which can be captured by pupillometry but not fully by EEG or fMRI. Consequently, the adoption of multiple and multimodal measurements and analysis techniques is anticipated to increase, to enhance our understanding of complex neural processes.

## Data Availability

The original contributions presented in the study are included in the article/supplementary material, further inquiries can be directed to the corresponding author.
